# Association between IL-17F, IL-17RA Gene Polymorphisms and Response to Biological Drugs in Psoriasis and Beyond

**DOI:** 10.3390/genes14051123

**Published:** 2023-05-22

**Authors:** Alexandra Dana Pușcaș, Iulia Ioana Morar, Ștefan Cristian Vesa, Andreea Cătană, Cristian Pușcaș, Roxana Flavia Ilieș, Remus-Ioan Orasan

**Affiliations:** 1 Department of Physiology, University of Medicine and Pharmacy Iuliu Hatieganu, 400347 Cluj-Napoca, Romania; rorasan@yahoo.com; 2 Department of Pathophysiology, University of Medicine and Pharmacy Iuliu Hatieganu, 400347 Cluj-Napoca, Romania; 3 Department of Pharmacology, Toxicology, and Clinical Pharmacology, University of Medicine and Pharmacy Iuliu Hatieganu, 400347 Cluj-Napoca, Romania; stefanvesa@gmail.com; 4 Department of Genetics, University of Medicine and Pharmacy Iuliu Hatieganu, 400347 Cluj-Napoca, Romania; catanaandreea@gmail.com (A.C.); roxanaflaviailies@gmail.com (R.F.I.); 5 Vadaskert Child and Youth Psychiatry Hospital, 1021 Budapest, Hungary; dr.pcristian@gmail.com

**Keywords:** psoriasis, rs763780, rs4819554, IL-17F, IL-17RA, biological treatment

## Abstract

Psoriasis is a systemic inflammatory disease that associates with multiple comorbidities. It involves complex interactions between environmental factors and polygenic predisposition. The IL-17 family is one of the main actors in the pathogenesis of psoriasis. Secondary nonresponse is common, especially during the long-term use of TNF-α inhibitors, but it is not uncommon even for newer biologics, such as IL-17 inhibitors. Identification of clinically useful biomarkers of treatment efficacy and safety would enable optimal treatment selection, improve patient quality of life and outcome, and reduce healthcare costs. To our knowledge, this is the first study to evaluate the relationship between genetic polymorphism of IL-17F (rs763780) and IL-17RA (rs4819554) and response to biological treatment and other clinical data in bio-naive and secondary non-responders psoriasis patients in Romania and Southeastern Europe. We performed a prospective, longitudinal, analytical cohort study of 81 patients diagnosed with moderate-to-severe chronic plaque psoriasis who received biological treatments for the first time. Of the 79 patients treated with TNF-α inhibitors, 44 experienced secondary nonresponse. All patients were genotyped for the two SNPs in IL-17F and IL-17RA genes. The rs763780 polymorphism in the IL-17F gene could be an attractive candidate biomarker for predicting which patients will respond to anti-TNF-α therapies. Another emergent association of rs4819554 in IL-17RA with the risk of nail psoriasis and a higher BMI in moderate-to-severe plaque psoriasis patients is described.

## 1. Introduction

In 2016, the World Health Organization (WHO) defined psoriasis as “a chronic, painful, disfiguring, and disabling non-communicable disease for which there is no cure” [1]. This systemic inflammatory disease may affect the skin, nails, and joints and is associated with multiple systemic complications [1,2,3,4]. Psoriasis affects 2–4% of the population worldwide [5], and the prevalence varies from 0.09% in Tanzania to 11.43% in Norway [6].

Currently, the pro-inflammatory cascade controlled by the IL-23/IL-17 axis is considered the most important element of the immunopathogenesis of psoriasis. IL-23 plays a role in the differentiation and activation of Th17 cells, which secrete mainly IL-17A and IL-17F, and by binding to its specific receptors, activates different cell types: keratinocytes, synoviocytes, fibroblasts, macrophages, B cells, or osteoclast precursors [7,8,9]. In the next phase, the inflammatory cells are recruited; these initiate and maintain inflammation during psoriasis and psoriatic arthritis [9]. The IL-17 family includes six members, noted from A to F, which bind to their five receptors noted from IL-17RA to IL-17RE [9,10,11,12]. IL-17A connects the innate and adaptive immune systems by mobilizing, recruiting, and activating neutrophils [13]. In the presence of bacteria and fungi, IL-17A induces chemokine expression by keratinocytes, induces the production of pro-inflammatory cytokines, and leads to an immune response in the skin [14]. It also works together with TNF-α to stimulate keratinocytes to produce pro-inflammatory cytokines [12,15]. IL-17F shares 50% homology with IL-17A and also causes the release of pro-inflammatory cytokines and mobilizes neutrophils [9,12,14]. IL-17A and IL-17F share the same receptor that consists of two units, IL-17RA and IL-17RC [16]. Therefore, in psoriasis lesions, the pro-inflammatory effects on keratinocytes and neutrophils are due to both members of the IL-17 family [10,17].

Up to date, four classes of biologics targeting inflammatory cytokines are used to treat psoriasis: TNF-α inhibitors; IL-12/23 inhibitors; IL-17 inhibitors; and IL-23 inhibitors [18]. It has not been possible to determine which biological agent is the best to use; therefore, several factors must be considered when choosing the biological therapy, including psoriasis phenotype, the presence of psoriatic arthritis, results obtained with previous systemic treatments, associated comorbidities, frequency or mode of administration, and aspects of personal life [19]. TNF-α inhibitors are the oldest agents approved for the treatment of psoriasis and psoriatic arthritis, and their long-term safety profile is well-known. The most effective TNF-α inhibitor is infliximab, followed by certolizumab, and adalimumab, with similar efficacies, while adalimumab has the lowest efficacy [20]. IL-17 inhibitors are also approved for the treatment of psoriasis and psoriatic arthritis. This biological class associates fast and very good response, and its members have comparable effectiveness due to their similar mechanism of action [21]. Genes that are synergistically regulated by both TNF-α and IL-17 are more successfully blocked by IL-17A antagonists than by anti-TNF-α agents [22]. More precisely, two weeks after the administration of 150 mg ixekizumab, 765 genes were normalized compared to less than 200 genes in the case of etanercept [22]. Although biologics have revolutionized the treatment of psoriasis, a primary or secondary nonresponse is reported in 20–50% of cases [17]. Secondary nonresponse is common, especially during the long-term use of TNF-α inhibitors, but it is not uncommon even for newer biologics, such as IL-17 inhibitors [23,24]. This phenomenon is more frequent than primary unresponsiveness and is associated with a gradual decline of efficacy after clinical remission is achieved during the first six months [18,24,25]. The need to identify biomarkers that predict the response to biological treatment is becoming increasingly necessary.

Most of the genes involved in psoriasis encode proteins related to the innate and adaptive immune systems, and a small number of genes encode skin proteins [5]. GWAS studies have identified more than 80 loci associated with the risk of developing psoriasis [2,3,4]. PSORS1, located on the chromosomal region 6p21, is the most well-known locus with the highest susceptibility for psoriasis [2,3,4]. The most studied allele is HLA-Cw6, which is present in approximately 60% of those diagnosed with psoriasis and increases the risk of disease by 9 to 23-fold [4]. There is a strong association between HLA-Cw6 and early onset, severe, and unstable forms of psoriasis [2,4,8,26]. The same chromosome (6p.12) hosts the IL-17A and IL-17F genes [9], while IL-17RA resides on chromosome 22 [10,17]. The rs763780 SNP is a missense mutation that affects transcriptional regulation and gene expression of IL-17F [27,28]. The rs4819554 SNP resides in the promoter region of the IL-17RA gene and affects gene transcription, which influences IL-17RA expression and determines subsequent biological effects [9,17]. In different populations, SNPs belonging to IL-17RA or IL-17F and other pro-inflammatory cytokines have been associated with the susceptibility to develop psoriasis or psoriatic arthritis and with the response to biological treatment. For example, rs763780 of IL-17F has been associated with response to infliximab and ustekinumab at 3 and 6 months of treatment and to adalimumab at 6 months in the Spanish population [14]. Several studies reported a strong association of this SNP in IL-17F with susceptibility to psoriasis [27,28,29,30,31]. Regarding rs4819554 of IL-17RA, Batalla et al. found a connection between this SNP and the response to anti-TNF agents at week 12 in a Spanish population [17]. Another study conducted by Sabry et al. demonstrated a connection between IL-17RA SNP with psoriasis risk in the Egyptian population [32]. Based on currently available data, IL-17F and IL-17RA gene variants have significant effects on psoriasis. The most studied IL-17F SNP is rs763780, and it is clear that this locus plays an important role in the human immune system [28]. A growing body of evidence would help scientists to understand the effects of certain alleles on the pathogenesis of psoriasis and clarify their exact impact in response to biological treatments.

Genetic studies have allowed the development of new biological drugs that are effective and safe, but variations in response to the prescribed therapy, toxicity, or loss of benefit over time are not uncommon. Genetic polymorphisms can affect the mechanism of action of drugs, thus explaining the variability in response to treatment [33]. Identification of clinically useful biomarkers of treatment efficacy and safety would enable optimal treatment selection, improve patient quality of life and outcome, and reduce healthcare costs [34].

Our study aims to identify whether the SNPs rs763780 (in the IL-17 gene) and rs4819554 (in the IL-17RA gene) are associated with the response to biological treatment or other data collected in the study.

## 2. Materials and Methods

### 2.1. Study Population

Our prospective, longitudinal, analytical cohort study included 81 patients diagnosed with moderate-to-severe chronic plaque psoriasis. The patients were selected between November 2016 and September 2017 and were followed up from the moment they were included in the study until January 2023. The study was approved by the Ethics Committee of the University of Medicine and Pharmacy “Iuliu Hatieganu,” Cluj-Napoca, Romania. All patients signed an informed consent before enrollment. The patients with chronic plaque psoriasis were recruited through the Dermatology Clinic of the County Emergency Hospital Cluj-Napoca.

Psoriatic patients met a series of inclusion criteria as follows: patients of both sexes; over the age of 18; the presence of moderate-to-severe plaque psoriasis (defined according to the European Consensus by Mrowietz) [35]; diagnosis based on clinical and histopathological examination; patients with no response to local and systemic classical treatments; and bio-naive patients who indicated biological treatment. The exclusion criteria consisted of the patients with other forms of psoriasis (due to their different genetic backgrounds), presence of other severe skin diseases (atopic dermatitis, pemphigus vulgaris, alopecia areata, dermatomyositis, or cutaneous lupus erythematosus), history of malignancy, history of asthma, ulcerative colitis, Crohn’s disease, rheumatoid arthritis, ankylosing spondylitis (because of their association with rs763780 in IL-17F or rs4819554 in IL-17RA) [36,37,38], pregnant or lactating females, death or discontinuation of the biological therapy during the follow-up period. All patients were Caucasian and were between 18 and 83 years old. During the follow-up period, four major classes of biological treatments were used: anti-TNF-α; anti-IL23; anti-IL17; and anti-IL-12/23. Some patients were non-responders to one or more biological agents. Two treatment groups were established, the first-line biological treatment group (bio-naive patients) and the second-line biological treatment group (patients who developed resistance to the first-line treatment and required a second biological agent).

First-line biological treatment included 79 patients treated with anti-TNF-α agents, one patient treated with an anti-IL17A agent (secukinumab), and another patient with an anti-IL12/23 agent (ustekinumab). Among those treated with anti-TNF-α agents, 5 patients required a change in treatment before 48 weeks.

Second-line biological treatment included 44 patients and was organized into three classes of biologic agents: anti-TNF-; anti-IL23 or anti-IL-12/23; and anti-IL17A. All these patients were non-responders to anti-TNF agents (infliximab, adalimumab, etanercept) administered as first-line therapy (secondary nonresponse). Due to the small number of patients who developed resistance to the first-line treatment, we could not perform statistical analysis for each agent of the used classes. Of these, 4 patients treated with anti-TNF-α and one treated with anti-IL17A required a change in treatment before 48 weeks.

Due to the small number of patients who developed more than two resistances to biological treatment, statistical analysis could not be performed in this group of patients. Thus, 14 patients required a third biological agent, 6 patients required a fourth agent, and one patient required a fifth agent.

The effectiveness of treatment was evaluated using the Psoriasis Area and Severity Index (PASI). The score was recorded at baseline, weeks 12, 24, 36, and 48 for each treatment. Responders to treatment were considered to be all the patients who achieved PASI75 (at least a 75% reduction from their baseline PASI) at week 24 and PASI90 (at least a 90% reduction from their baseline PASI) at week 48. Additionally, we evaluated the percentage reduction in baseline PASI at 12, 24, 36, and 48 weeks. Blood samples taken during the first examination were stored at −20 °C until they were sent to the genetic laboratory at the Medical Genetics Department of the University of Medicine and Pharmacy “Iuliu Hatieganu”, Cluj-Napoca, Romania, to genotype for IL-17RA rs4819554 and rs763780 IL-17F.

For each patient included in the study, we also recorded BMI, smoking or alcohol consumption, presence or absence of associated comorbidities (type 2 diabetes, hypertension, cardiovascular diseases, dyslipidemia), PsA, or nail psoriasis.

### 2.2. Polymorphisms Studied

We selected two single nucleotide polymorphisms (SNPs), rs763780 in the IL17F gene and rs4819554 in the IL17RA gene. These SNPs were previously associated with response to biological treatment or the risk of developing psoriasis in other populations [10,14,27,28,31,32,39,40].

### 2.3. DNA Isolation and Genotyping

Genomic DNA was extracted using Wizard Genomic Purification Kit (Promega, Madison, MI, USA) from 400 µL venous blood samples. NanoDrop 2000/2000c spectrophotometer (ThermoScientific, Waltham, MA, USA) was used to evaluate the concentration and purity of DNA. TaqMan 5′ nuclease allelic discrimination technology on the 96-well ABI 7900HT Real-Time PCR System (Applied Biosystems, Foster City, CA, USA) and SDS software (version 2.3) was used for genotype IL-17RA G197A (rs4819554) and IL-17F A7488G (rs763780) polymorphisms. For the PCR reaction, a total volume of 20 μL reaction mixture containing DNAase-free water, 10 µL TaqMan Universal PCR Master Mix (Applied Biosystems), 20 ng DNA, 1 µL of TaqMan Applied Biosystem SNP Genotyping Assay 20× mix (assay ID C__337392_30 for rs4819554 and C__2666446_20 for rs763780) were used. For thermal cycling conditions, the following steps were followed: initial AmpliTaq Gold enzyme activation at 95 °C for 10 min; 35 denaturation cycles at 95 °C for 15 s; and annealing/extension for 1 min at 60 °C. Moreover, 6-carboxy-X-rhodamine (ROX) was used as a passive reference dye.

### 2.4. Statistical Analysis

The statistical analysis was performed using MedCalc^®^ Statistical Software version 20.2.18 (MedCalc Software Ltd., Ostend, Belgium; https://www.medcalc.org; accessed on 25 April 2023). The data were assessed for normality using the Shapiro–Wilk test. Means and standard deviations were used to describe continuous variables, while frequencies and percentages were used to describe qualitative data. Two-way ANOVA for repeated measurements was used to compare differences between two groups at different times. To assess the association between categorical variables, the chi-square test was used. A *p*-value of <0.05 was considered statistically significant.

## 3. Results

### 3.1. Description of Our Population

The group had a mean age of 50 ± 13.9 years, and the mean age at the onset of the disease was 33.7 ± 15.9 years. Table 1 summarizes the demographic data, clinical data, and genotype distribution.

The correlation between disease severity and sex, BMI, alcohol consumption, and smoking did not reach statistical significance (Table 2).

However, those who were overweight or obese had a slightly higher PASI score compared to those with normal weight. Among those diagnosed with nail psoriasis, 41.3% also had concomitant PsA.

In our study group, 22.2% of the patients reported frequent alcohol consumption; however, we found no significant difference in the mean baseline PASI between alcohol consumers and non-consumers. Additionally, we observed that smoking did not have an impact on the severity of the disease in our study group.

The first-line biological treatment administered was an anti-TNF-α agent (infliximab, adalimumab, or etanercept) to 79 patients, as shown in Table 3. Out of these patients, 5 required a change in treatment before 48 weeks. The percentage of improvement was 75.92% ± 10.97 at 24 weeks and 92.08% ± 11.27 at 48 weeks. Patients treated with infliximab exhibited treatment resistance after a median of 36 months, followed by those treated with Etanercept at a median time of 66 months and Adalimumab at 67 months.

The group of 44 patients treated with a second-line biological treatment consisted of anti-TNF-α, anti-IL-23 or anti-IL-12/23, and anti-IL-17A agents, as presented in Table 4. The percentage of improvement was 81.27% ± 10.84 for anti-TNF-α agents, 86.55% ± 15.99 for anti-IL23 or anti-IL-12/23, and 85.89% ± 11.78 for anti-IL-17A at 24 weeks, respectively, and 89.51% ± 14.9, 96.92% ± 7.12, and 91.21% ± 21.65 at 48 weeks.

### 3.2. Association between SNPs and Response to Treatment: First-Line Treatment

At week 24, 52 (65.8%) of biologically-naive patients treated with anti-TNF-α were responders, having achieved PASI75, and 61 (82.4%) of them achieved PASI90 by week 48. The highest response rate at week 24 was seen in those treated with etanercept, while infliximab had the highest response rate at week 48, with etanercept showing the poorest response rate. However, when we analyzed all biological agents together, we did not observe any relationship between rs763780 IL-17F or rs4819554 IL-17RA and response to treatment.

**Figure 1:**
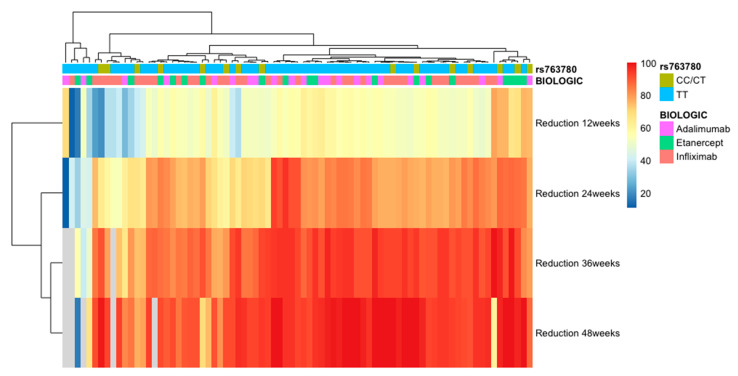
Heatmap of association between response to anti-TNF-α agents (adalimumab, etanercept, infliximab) and rs763780 in IL-17F gene, TT, and CC/CT genotypes. Response to treatment was evaluated by a percentual reduction in the baseline PASI score at 12, 24, 36, and 48 weeks.

**Figure 2:**
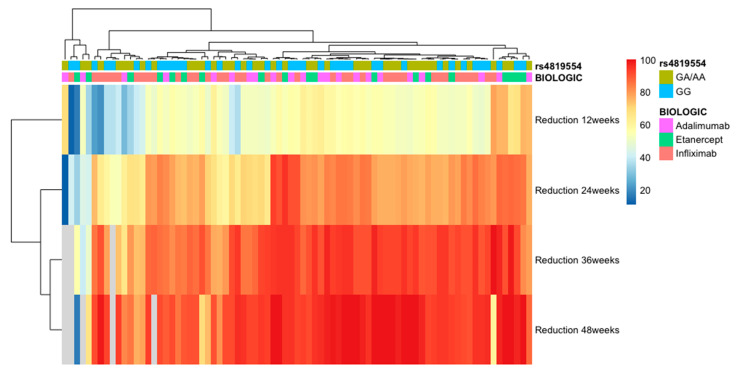
Heatmap of the lack of association between response to anti-TNF-α agents (adalimumab, etanercept, infliximab) and rs4819554 in IL-17RA gene, GG, and GA/AA genotypes. Response to treatment was evaluated by a percentual reduction in the baseline PASI score at 12, 24, 36, and 48 weeks.

When we analyzed each anti-TNF-α agent separately, we noticed that rs763780 IL-17F was associated with response to infliximab and adalimumab treatment (*p* = 0.04 and *p* = 0.0001, respectively) when evaluating the response to treatment by the percentage reduction in baseline PASI. Thus, in the case of infliximab-treated patients, the TT genotype was associated with an improved response to treatment at 12 weeks, while the CT/CC genotype was associated with an improved response at 36 and 48 weeks. In patients treated with adalimumab, the CT/CC genotype was associated with a good response at 12 weeks, while those with the TT genotype were more likely to respond favorably at 36 and 48 weeks (see Figure 1). There was no association between patients treated with infliximab or adalimumab who reached PASI75 or PASI90 and the rs763780 IL-17F polymorphism. In our study, this polymorphism was not associated with the response to etanercept treatment (see Table 3). Regarding the rs4819554 polymorphism, we found no association with the response to infliximab, etanercept, or adalimumab treatment (see Table 3 and Figure 2).

### 3.3. Association between SNPs and Response to Treatment: Second-Line Treatment (Table 4)

No statistical association was found between response to TNF-α inhibitors and rs763780 IL-17 (*p* = 0.07, *p* = 0.09 respectively) or rs4819554 IL-17RA at 24 and 48 weeks. The response to anti-TNF-α treatment was evaluated using the percentage reduction in the baseline PASI score at 12, 24, 36, or 48 weeks and no statistical association with the studied polymorphisms (*p* = 0.08 for IL-17F SNP) was found. As we can observe, the *p* values obtained in the case of IL-17F SNP are not too far from the significant statistical value.

In the groups of those treated with anti-IL17A and anti-IL23 or anti-IL12/23, those who were responders at 24 weeks maintained the same status at 48 weeks. The highest percentage of responders was in the group of those treated with anti-IL-17A agents (89.5%). In both groups, all patients with GA/AA genotype of rs4819554 IL-17RA were responders at 24 and 48 weeks. Patients treated with anti-IL23 or anti-IL12/23 agents show no association between polymorphisms and response to treatment, neither in the case of the percentage reduction in the baseline PASI nor in the case of those reaching PASI75 at 24 weeks or PASI90 at 48 weeks. Moreover, patients treated with anti-IL17A agents show no association with our studied polymorphism in none of the previously mentioned cases.

### 3.4. Association between SNPs and Clinical Characteristics: The Entire Study Group (Table 1)

Polymorphism rs4819554 of the IL-17RA gene is more frequently associated with nail psoriasis in patients with psoriasis who have the GG genotype compared to those with GA/AA genotype (*p* = 0.02). We also found an association between the IL-17RA SNP and BMI. A allele carriers are more likely to be overweight, while those with the GG genotype are more likely to be obese (*p* = 0.05). No significant relationships were observed between rs763780 IL-17F and rs4819554 IL-17RA and other clinical data collected in this study.

## 4. Discussion

Currently, the IL23/Th17 axis plays a crucial role in the occurrence and development of psoriasis, especially due to the IL-17A and IL-17F proinflammatory cytokines, which are among the most often incriminated factors in pathogenesis. Few studies on the rs763780 in IL-17F and rs4819554 in IL-17RA gene polymorphisms in psoriasis have been reported until now [14,17,41]. We examined the relationship between two SNPs in genes encoding IL-17RA and IL-17F and their response to biological agents. In our study, in bio-naive patients, response to infliximab and adalimumab was associated with rs763780 IL-17F, although there was no association with etanercept. For those with the TT genotype, the response to infliximab treatment registered a better outcome for the first 12 weeks (short-term positive response). Later on, the CT/CC genotype was associated with a greater percentage reduction in PASI, with the most significant improvement between 24 and 36 weeks and an even more substantial reduction at 48 weeks. In contrast, the response to adalimumab treatment was greater in the first 12 weeks for those with the CT/CC genotype, while the TT genotype was associated with a better response at 36 and 48 weeks (long-term positive response).

Almost similar results were reported by Prieto-Perez et al. in the Spanish population. The studied SNP in the IL-17F gene showed that the CT genotype was associated with response to infliximab at 12–16 and 24–28 weeks of treatment, and the TT genotype was associated with adalimumab response at 24–28 weeks [14]. The same study reported no association of this SNP with response to etanercept treatment [14]. This can be explained by the fact that infliximab and adalimumab exert effects on the transmembrane TNF-α of activated immune cells, while etanercept targets soluble TNF-α [42]. Rs763780 in the IL-17F gene affects the transcriptional regulation and gene expression of its homologous interleukin [27]. Kawaguchi et al. studied this SNP in vitro and concluded that a reduced expression and activity of IL-17F was linked to a mutant C allele, which caused the mutant IL-17F to lose its ability to activate the production of chemokines and proinflammatory cytokines, such as TNF-α [37]. Responders to anti-TNF-α agents associate suppression of Th17 signaling pathways, which have an inhibitory effect on the signaling pathways of IL-17 [43]. Most likely, this mutant IL-17F interferes with the inflammatory response and also prevents the physiological interaction with its receptor [37]. Non-responders carrying the C mutant allele in rs763780 may have a reduced immune response due to this abnormal IL-17F, which could explain why anti-TNF-α agents lose their effectiveness [14]. In our study, carriers of the C allele in rs763780 were poor responders to adalimumab at 36 and 48 weeks. This is also confirmed in the Spanish population, where those carrying the C allele were also poor responders to adalimumab at 24–28 weeks [14]. Other studies conducted on patients suffering from rheumatoid arthritis or ankylosing spondylitis suggest an association between response to anti-TNF-α agents and the IL-17F SNP [44,45].

We couldn’t establish a relationship between rs4819554 in IL-17RA and response to treatment, but we report two associations regarding the susceptibility to develop nail psoriasis and increased BMI in patients with moderate-to-severe psoriasis. Nail psoriasis affects around 50% of patients and can be an indicator of PsA [46]. In general, the treatment is not chosen depending on the presence or absence of nail damage, but biologics are reported to be effective for this problem, as well [46,47]. If the goal is to primarily treat nail psoriasis, then the best option is ixekizumab [46], which underlines the importance of IL-17 in the process of nail psoriasis. Obesity prevalence is higher in psoriasis patients, and it is well-recognized as a risk factor for this disease [26,48]. Obese patients tend to associate a more severe form of psoriasis, and weight reduction may improve the course of the disease [26,48,49,50]. Obesity is linked to a higher grade of systemic inflammation; adipocytes are involved in the production of IL-6, which contributes to the differentiation of T lymphocytes into the Th17 subtype [50]. The most important IL-17-producing cells are Th17 [51], thus explaining the elevated levels of IL-17 in obese patients [50]. Regarding our mentioned associations, we did not find any similar reports in the literature; however, our results should be confirmed in larger sample size groups. Regarding the response to treatment, Batalla et al. reported an association between this SNP of IL-17RA, AA genotype, and response to anti-TNF-α agents at week 12 [17]. This discrepancy between the Spanish and Romanian populations can be explained by genetic heterogeneity and also socioeconomic or other environmental factors.

Additionally, no association was found between the two studied polymorphisms and the response to biologic classes used as second-line therapy in patients with secondary nonresponse to anti-TNF-α agents. On the other hand, other studies have found no association between SNPs in IL-17F and the response to secukinumab in the Turkish population [41] or topical and/or NB-UVB treatment in the Polish population [39]. No results regarding the two studied polymorphisms and the response to anti-IL-23 or anti-IL-12/23 in psoriasis patients were available in the literature.

The present study was based on a limited number of patients diagnosed with moderate-to-severe psoriasis vulgaris, and this probably contributed to the lack of some associations between the studied SNPs and the response to treatment, although valuable results were obtained. This could explain the lack of association between the rs763780 TT genotype with responder status (PASI75) for infliximab at week 24, where we obtained a *p*-value of 0.08, which was close to statistical significance. A larger study group may confirm an association for these variables. Likewise, three patients treated with infliximab needed a change in biological agent between 24–36 weeks of treatment, and all these patients were TT genotype, which most likely influenced the lack of association with the response to treatment at week 48 (PASI90). Another example, for the same SNP in IL-17F, is from the second-line therapy group in the case of those treated with anti-TNF-α, where we obtained *p*-values close to statistical significance, as follows: *p* = 0.08 when evaluating the percentage reduction in baseline PASI score at 12, 24, 36, and 48 weeks; *p* = 0.07 for responders at 24 weeks (PASI75); or *p* = 0.09 in the case of responders at 48 weeks (PASI90). In all these cases, in a larger group, the TT genotype probably would be associated with a better response to this treatment, once more confirming the potential role of rs763780 IL-17F as a predictive biomarker for the response to anti-TNF-α agents. Regarding those treated with anti-IL17A and anti-IL23 or IL-12/23 (also small sample size groups in our study), in larger groups, there could be a possible association between rs4819554 with the response to these treatments in patients carrying the A allele. In addition, other limitations of our study were as follows: few published studies on the IL-17F rs763780 and IL-17RA rs4819554 polymorphisms and their impact on biological treatment in patients with psoriasis; the lack of NAPSI score registration at baseline and in evolution, so we could not evaluate the genotype effect on the severity of the nail damage or the response to treatment.

Finally, this study was based on patients from a single population. Therefore, further studies are needed to confirm our results in larger cohorts of multiethnic populations and groups of patients treated with newer biological drugs, to understand how the effects of these SNPs vary based on race and chosen treatment. We suggest that SNP rs763780 in IL-17F, through its effects on IL-17F, has an important role in the pathogenesis of psoriasis. Hence, it should be considered as a possible useful clinical marker for anti-TNF-α treatment outcomes in patients with moderate-to-severe plaque psoriasis. To our knowledge, the present study is the first to investigate these associations in our population.

Across medicine, including psoriasis, studies have reported candidate biomarkers of efficacy to biological treatments. A systematic review published by Corbett et al. identified candidate biomarkers of efficacy to anti-TNF-α drugs and ustekinumab [34]. Yet, none of them can be used as clinical biomarkers due to insufficient data, and further studies are needed for their validation [34]. Most likely, in the near future, these biomarkers will become available for clinical use and will have a positive and major impact on the management of psoriasis.

## 5. Conclusions

We report an association between the rs763780 polymorphism in the IL-17F gene and the response to infliximab and adalimumab treatment at weeks 36 and 48. Another important and interesting finding is the association of rs4819554 in IL-17RA with the risk of nail psoriasis and a higher BMI in moderate-to-severe plaque psoriasis patients. This is the first study to evaluate these two polymorphisms in bio-naive and secondary non-responders patients with moderate-to-severe plaque psoriasis in Romania and southeastern Europe. We encourage further investigation of these associations in larger study populations to clarify the role of these SNPs in gene expression and clinical approach. Genetic polymorphisms are known to interfere with the mechanism of action of drugs [33] and can explain the variability in response to biological treatments. The rs763780 polymorphism in the IL-17F SNP could be an attractive candidate biomarker for predicting which patients will respond to anti-TNF-α therapies and which will not.

## Figures and Tables

**Table 1:** Demographic, clinic, and genotype data.

*n* = 81	rs763780 IL-17F		*p*	Total	rs4819554 IL17RA		*p*
	TT	CT/CC		*n* = 81	GG	GA/AA	
Male	45 (81.8%)	10 (8.2%)		55 (67.9%)	28 (50.9%)	27 (49.1%)	
Female	19 (73.1%)	7 (26.9%)		26 (32.1%)	11 (42.3%)	15 (57.7%)	
PASI baseline (mean ± s.d. *)	23.2 ± 6.1	23.9 ± 9.2	0.9	23.3 ± 6.9	23.4 ± 6.8	23.2 ± 7	0.9
PsA	18 (28.1%)	9 (52.9%)	0.1	27 (33.3%)	16 (41%)	11 (26.2%)	0.2
Nail psoriasis	21 (32.8%)	8 (47.1%)	0.4	29 (35.8%)	19 (48.7%)	10 (23.8%)	0.02 *
Hypertension	27 (42.2%)	7 (41.2%)	1	34 (42%)	18 (46.2%)	16 (38.1%)	0.6
C-v diseases **	11 (17.2%)	0 (100%)	0.1	11 (13.6%)	6 (15.4%)	5 (11.9%)	0..8
Diabetes type 2	12 (18.7%)	2 (11.7%)	0.7	14 (17.2%)	8 (20.5%)	6 (14.2%)	0.6
Dyslipidemia	25 (39.1%)	7 (41.2%)	0.6	32 (39.5%)	16 (41%)	16 (38.1%)	0.6
Normal weight	20 (31.3%)	3 (17.6%)	0.4	23 (28.4%)	11 (28.2%)	12 (28.6%)	0.05 *
Overweight	22 (34.4%)	6 (35.3%)		28 (34.6%)	9 (28.2%)	19 (45.2%)	
Obesity	22 (34.4%)	8 (47.1%)		30 (37%)	19 (48.7%)	11 (26.2%)	

* s.d.- standard deviation. ** c-v diseases- cardiovascular diseases.

**Table 2:** Severity of the disease vs. sex, IMC, and alcohol/smoking.

*n* = 81	PASI Baseline (Mean ± s.d.)	*p*
Male	23.2 ± 6.2	0.7
Female	23.7 ± 8.2	
Normal weight	22.5 ± 6.8	0.5
Overweight	23 ± 6.2	
Obese	24.4 ± 7.6	
Alcohol *n* = 18 (22.2%)	23.8 ± 5.2	0.7
Smoking *n* = 26 (32.1%)	23.8 ± 7.2	0.7

**Table 3:** First-line treatment: patients treated with anti-TNF-α agents and treatment outcomes.

*n* = 79	IL-17F rs763780					IL-17RA rs4819554				
	TT		CT/CC		*p*	GG		GA/AA		*p*
INFLIXIMAB (*n* = 38)										
%reduction * 12 weeks	51.1 ± 7.4		42 ± 12.4		**0.04** *	49.1 ± 9.3		49.6 ± 9.2		0.5
%reduction 24 weeks	76.1 ± 9.6		67.3 ± 8.5			75.5 ± 10.7		72.7 ± 8.8		
%reduction 36 weeks	88.9 ± 6.3		85.5 ± 8.3			88.7 ± 7.1		87.5 ± 6.5		
%reduction 48 weeks	93.1 ± 6.4		92.3 ± 7.7			92.4 ± 7.4		93.7 ± 5.3		
*n*	28		7			20		15		
	Yes	No	Yes	No		Yes	No	Yes	No	
PASI75	67.7%	32.2%	28.6%	71.4%	*0.08*	52.4%	47.6%	70.6%	29.4%	0.4
*n*	21	10	2	5		11	10	12	5	
PASI90	85.7%	14.3%	85.7%	14.3%	1	80%	20%	93.3%	6.7%	0.3
*n*	24	4	6	1		16	4	14	1	
ADALIMUMAB (*n* = 22)										
%reduction 12 weeks	53.1 ± 8.1		75 ± 0.6		**0.0001** *	58.1 ± 7.8		53.1 ± 11.6		0.6
%reduction 24 weeks	78.4 ± 8.2		81.8 ± 7.3			81.5 ± 6		76.4 ± 8.9		
%reduction 36 weeks	88.7 ± 7.2		87.9 ± 12.4			90.9 ± 4.2		86.8 ± 9.1		
%reduction 48 weeks	94.7 ± 4.5		92.1 ± 6.5			95.1 ± 3.1		93.9 ± 5.6		
*n*	18		2			9		11		
	Yes	No	Yes	No		Yes	No	Yes	No	
PASI75	65%	35%	100%	0%	1	88.9%	11.1%	53.8%	46.2%	0.1
*n*	13	7	2	0		8	1	7	6	
PASI90	88.9%	11.1%	50%	50%	0.2	88.9%	11.1%	81.8%	18.2%	1
*n*	16	2	1	1		8	1	9	2	
ETANERCEPT (*n* = 19)										
%reduction 12 weeks	53.7 ± 16.5		56 ± 6.1		0.8	54 ± 17		54.8 ± 12		0.7
%reduction 24 weeks	74.2 ± 17.5		79.1 ± 5.5			74.7 ± 17.2		76.5 ± 13.5		
%reduction 36 weeks	83.9 ± 15.1		90.9 ± 2.8			83.5 ± 12.5		87.9 ± 13.4		
%reduction 48 weeks	86.4 ± 22.8		91.1 ± 10.9			85 ± 27.8		90 ± 11.6		
*n*	13		6			8		11		
	Yes	No	Yes	No		Yes	No	Yes	No	
PASI75	69.2%	30.8%	83.3%	16.7%	1	75%	25%	72.7%	27.3%	1
*n*	9	4	5	1		6	2	8	3	
PASI90	69.2%	30.8%	83.3%	16.7%	1	75%	25%	72.7%	27.3%	1
*n*	9	4	5	1		6	2	8	3	

* % reduction—percentual reduction in the PASI score.

**Table 4:** Second-line treatment: patients treated with anti-TNF-α agents; anti-IL-23 or anti-IL-12/23; anti-IL-17A; treatment outcomes.

*n* = 44	IL-17F rs763780				*p*	IL-17RA rs4819554				*p*
	TT		CT/CC			GG		GA/AA		
ANTI-TNF-α (*n* = 12)										
%reduction * 12 weeks	63.2 ± 16.9		23 ± 4.1		*0.08*	59.1 ± 24.5		51.4 ± 17.2		0.4
%reduction 24 weeks	84.3 ± 8.9		65.7 ± 1.2			80.1 ± 10.6		83.4 ± 12.5		
%reduction 36 weeks	93 ± 10.1		70.2 ± 10.3			89.2 ± 11.5		89.1 ± 17.2		
%reduction 48 weeks	93.6 ± 10.3		68.8 ± 21.4			90.8 ± 11.4		86.8 ± 22.3		
*N*	10		2			8		4		
	Yes	No	Yes	No		Yes	No	Yes	No	
PASI75	81.8%	18.2%	0%	100%		75%	25%	60%	40%	
*N*	9	2	0	2	*0.07*	6	2	3	2	1
PASI90	80%	20%	0%	100%		62.5%	37.5%	75%	25%	
*N*	8	2	0	2	*0.09*	5	3	3	1	1
ANTI-IL23 OR ANTI-IL12/23 (*n* = 13)										
%reduction 12 weeks	59.9 ± 12.9		63.2 ± 21.1		0.4	56.7 ± 17.1		65.3 ± 9.1		0.8
%reduction 24 weeks	87.6 ± 17		82.8 ± 14.2			83.5 ± 20.8		90 ± 8.1		
%reduction 36 weeks	96.1 ± 11.8		92.5 ± 12.8			91.2 ± 15		100 ± 0		
%reduction 48 weeks	97.9 ± 5.8		93.5 ± 11.2			94.2 ± 9.1		100 ± 0		
*n*	10		3			7		6		
	Yes	No	Yes	No		Yes	No	Yes	No	
PASI75	90%	10%	66.7%	33.3%		71.4%	28.6%	100%	0%	
*n*	9	1	2	1	0.4	5	2	6	0	0.4
PASI90	90%	10%	66.7%	33.3%		71.4%	28.6%	100%	0%	
*n*	9	1	2	1	0.4	5	2	6	0	0.4
ANTI-IL-17A (*n* = 19)										
%reduction 12 weeks	67.2 ± 15.9		57.2 ± 30.6		0.6	58.9 ± 29		67.1± 15.9		0.2
%reduction 24 weeks	87.5 ± 8.4		82.3 ± 17.5			81.2 ± 16.3		88.6 ± 7.7		
%reduction 36 weeks	92.8 ± 17.6		87.5 ± 18.5			81.4 ± 26.8		96.8 ± 4.3		
%reduction 48 weeks	91.5 ± 23.4		90.5 ± 19			79.6 ± 33.6		97.9 ± 3.8		
*N*	13		6			7		12		
	Yes	No	Yes	No		Yes	No	Yes	No	
PASI75	92.3%	7.7%	83.3%	16.7%		71.4%	28.6%	100%	0%	
*n*	12	1	5	1	1	5	2	12	0	0.1
PASI90	92.3%	7.7%	83.3%	16.7%		71.4%	28.6%	100%	0%	
*n*	12	1	5	1	1	5	2	12	0	0.1

* % reduction—percentual reduction in the PASI score.

## Data Availability

Not applicable.

## Abbreviations

The following abbreviations are used in this manuscript:

IL	Interleukin
SNP	Single nucleotide polymorphism
BMI	Body mass index
TNF-α	Tumoral necrosis factor-α
GWAS	Genome-wide association study
PASI	Psoriasis area severity index
PsA	Psoriatic arthritis
